# Physiological and Molecular Responses of Underutilized Genotype AHK-200 of Vegetable Melon (*Cucumis melo* var. *melo*) Against Drought Stress: Gas Exchange, Antioxidant Activity, and Gene Expression

**DOI:** 10.3390/metabo15060359

**Published:** 2025-05-28

**Authors:** Sudhakar Pandey, Waquar Akhter Ansari, Ram Krishna, Akhilesh Yadav, Durgesh Kumar Jaiswal, Bijendra Singh

**Affiliations:** 1 Indian Council of Agricultural Research, Indian Institute of Vegetable Research, P.O.-Jakhani (Shahanshahpur), Varanasi 221305, Uttar Pradesh, India; mbt.r.krishna@gmail.com (R.K.); bsinghiivr@gmail.com (B.S.); 2 Indian Council of Agricultural Research, Pusa Campus, New Delhi 110012, India; 3 Marwadi University Research Centre, Department of Agriculture, Faculty of Science, Marwadi University, Morbi Road, Rajkot 360003, Gujarat, India; waquar.ansari@marwadieducation.edu.in; 4 Department of Plant Science, University of California, Davis, CA 95616, USA; 5 Department of Biotechnology, Graphic Era (Deemed to be University), Dehradun 248002, Uttarakhand, India; durgesh.jaiswal9@gmail.com

**Keywords:** drought, photosynthesis, antioxidant enzyme, gene expression, vegetable melon

## Abstract

**Background/Objectives**: Drought stress is a significant environmental challenge that affects plant growth and productivity. **Methods**: In this study, an underutilized and better drought stress tolerance genotype of *Cucumis melo* var. *melo*, i.e., AHK-200, was investigated for drought tolerance potential, with special emphasis on various morphological, physiological, biochemical, and molecular parameters. **Results**: Our findings show that AHK-200 demonstrates superior drought tolerance with an enhanced root length, better water retention capacity, and stable cell membrane integrity under water deficit conditions. Physiologically, AHK-200 exhibited minimal reduction in relative water content (RWC) and photosynthetic efficiency (*PN*), along with increased stomatal conductance (*gs*) and chlorophyll content and reduced photoinhibition under drought stress. Biochemically, AHK-200 showed higher antioxidant enzyme activity (APX, CAT, SOD, GR, POD) and osmolyte accumulation (proline), which are critical for mitigating oxidative stress. At the molecular level, drought-related genes such as *DREB2C*, *DREB2D*, and *RD22* were upregulated, supporting AHK-200 resilience to drought stress. Additionally, AHK-200 displayed elevated mineral concentrations, including Na, K, Ca, and Fe, which are essential for cellular homeostasis and stress adaptation. **Conclusions**: Overall, our study provides a comprehensive understanding of the drought tolerance mechanisms in AHK-200, highlighting its potential for use in breeding drought-tolerant genotypes in cucurbits and related crops. This research could guide future efforts in gene manipulation and transgenic development aimed at enhancing drought resistance and yield potential in crop plants. Furthermore, *DREB2C*, *DREB2D*, and *RD22* transcription factors regulate many pathways related to stress; the overexpression of these genes may open a new avenue in melon improvement against drought stress.

## 1. Introduction

In nature, large numbers of plant genetic resources exist that have region-specific adaptations and great adaptive potential against biotic and abiotic stresses. They can be utilized in breeding programs for the development of biotic and abiotic stress-tolerant genotypes. The genotype will facilitate food and nutritional security in the changing climate [1]. Among cucurbit crops, vegetable melon, locally called kachri melon (*Cucumis melo* var. *melo*), has been explored far less to date, although it has great potential to tolerate the abiotic stresses most associated with drought and heat stress with no or very little effect on productivity. Very few reports are available that show its yield and growth performance properties under normal and stressed environmental conditions [2]. Earlier, the Indian Council of Agriculture Research—Central Institute for Arid Horticulture (ICAR-CIAH), Bikaner, characterized many vegetable melon genotypes based on their various horticultural traits. Under heat stress conditions, they found that AHK-200 performed better under stress conditions, even with better productivity, making it a suitable material for breeding purposes to develop drought/heat-tolerant genotypes in different cucurbit crops [3]. AHK-200 fruits are 100–120 g in weight and are harvested at the age of 65–70 days after sowing. Single plants, on average, bear 20–25 fruits, with a yield capacity of 115–120 q/ha. The fruits, with a unique taste after ripening, are eatable, although unripened fruits can also be used as vegetables.

In the northwestern region of India, this fruit is mostly cultivated with regular farming methods. The northwestern part of India mostly faces extremely high temperatures, and irregular rainfall results in drought stresses, which limit farming with crops that are susceptible to such environmental constraints. Hence, farming mainly by utilizing native plant species would be a problem-solving approach under environmental constraints and, in many cases, resource limitations. In such circumstances, the native cucurbit crop might play a significant role, as it can be utilized in breeding programs for crop improvement for sustainable environments experiencing mainly drought and heat. It is possible to develop genotypes with the desired properties for greater economic significance at low cost and with better productivity [3,4].

Cultivation of vegetable melonin this region provides nutrition-rich food products and supports local diets in tribal and desert areas [5]. Surveys conducted by ICAR-CIAH from 1994 to 2004 revealed that cucurbit types in hot, arid regions exhibit distinct characteristics due to their eco-adaptation and climatic variability [6,7]. These drought-hardy cultivars demonstrate high productivity under abiotic stress conditions; however, the quality of marketable fruits varies significantly, leading to uncertainties in yield components across different production sites [4,8,9].

Traditionally, vegetable melon is cultivated in dunes and as a mixed crop in Nagaur, Jodhpur, Jaisalmer, Churu, Barmer, and Bikaner, where vegetable melon plants show vigorous growth and development. The AHK-200 genotype has become popular among farmers because of its superior horticultural traits, such as uniform harvests, early maturity, and short growth duration. India has approximately 38.70 million hectares of arid regions, which are spread across the states of Andhra Pradesh, Haryana, Rajasthan, Punjab, Gujarat, and Karnataka. The key characteristics of this area are low and erratic rainfall (with a coefficient of variation between 40% and 70%), extreme temperatures (1 °C to 48 °C), high wind velocity, sandy soils, and inhospitable climatic conditions. These factors limit agricultural practices to specialized fruit plants that are well-adapted to climatic, edaphic, and biotic adversities. Plants exhibiting adaptation to biotic or abiotic stresses possess complex mechanisms observable at morphological, physiological, biochemical, and genetic levels through changes in gene expression.

While AHK-200 has been characterized horticulturally, the underlying biological mechanisms contributing to its abiotic stress tolerance remain unexplored [2]. The current objective of this study is to explore the impacts of varying levels of drought stress on AHK-200 plants while examining the morphological, physiological, biochemical, and molecular changes. This research, with its potential to enhance crop resilience and reduce production costs, is a crucial step toward ensuring food availability and economic prosperity in the face of increasing climatic challenges. The identification of important traits, such as root vigor architecture, improved photosynthetic efficiency, and the upregulation of stress-responsive genes, will help to develop stress-tolerant cultivars.

## 2. Materials

### 2.1. Materials and Treatments

The vegetable melon genotype AHK-200 was selected for this study based on preliminary screening performed by [2] under field conditions. The experiment took place at the ICAR—Indian Institute of Vegetable Research in Varanasi, located at 25.10° N latitude and 82.52° E longitude. The plant’s cultivation was carried out in 10 L pots with dimensions of 24 cm in height and 20 cm in diameter. The experimental design included four treatment sets, which included a control group with no water deficit (0 days without water) and three different sets subjected to 7, 14, and 21 days of water deficit (DWD). Every treatment had three replications, and each replication consisted of five pots. The potting soil pH was 6.8, the organic carbon content was 0.39% (*w/w*), the available N content was 0.30% (*w/w*), the available P was 0.51 mg g^−1^, and the available K was 0.35 mg g^−1^. During the experimental period, the minimum and maximum mean temperature fluctuated between 22.14 °C and 39.2 °C, and relative humidity varied from 58% to 87%, with a 14 h photoperiod and a daily maximum irradiance of 400 µmol m^−2^ s^−1^. The greenhouse conditions were chosen to control the environmental factors and ensure uniform growth conditions. Watering was administered at a rate of 2 L every three days for each pot until the imposition of drought stress, which began 30 days from the date of germination. Drought stress was implemented by stopping irrigation for the specified durations: 0 days (control) and 7, 14, or 21 days. To do this, we first stopped irrigation for the 21 DWD plants, then, after 1 week, for the 14 DWD plants, and after 2 weeks, for the 7 DWD plants, and the control, kept as 0 DWD, was maintained with regular irrigation, and on the same day as 21 DWD, sampling from all the treatments and the other data recordings were performed.

For biochemical measurements, fully expanded leaves from the sixth or seventh node were harvested at intervals corresponding to the control and each drought treatment duration (0, 7, 14, and 21 DWD) and stored at −80 °C until analysis. Fresh leaves were used for other observations, with three samples taken from each genotype per replication to ensure statistical robustness in the results. This comprehensive experimental design, coupled with meticulous data collection and analysis, has provided valuable insights into these muskmelon genotypes’ physiological and biochemical responses under varying water deficit conditions. These findings are significant for understanding and enhancing the drought tolerance mechanisms of muskmelon plants, thereby contributing to the advancement of agricultural science and plant physiology.

### 2.2. Shoot Weight Measurement

Three representative plants from each genotype were carefully and meticulously extracted from their pots, ensuring minimal disturbance to the roots. This careful approach was taken to maintain the accuracy of the data and to provide a reliable basis for our research.

### 2.3. Root Length and Dry-to-Fresh Mass Ratio

Plants were extracted intact from the roots at 0, 7, 14, and 21 DWD to evaluate root length. After separation, the fresh mass (FM) of the shoots, roots, and leaves was recorded. Furthermore, these parts were dried at 80 °C in an oven for 48 h for dry mass (DM) measurements. The dry-to-fresh mass ratio of each portion was calculated as a percentage using formulas, which were adapted from Kausar [10] with slight modifications [11].

### 2.4. Net Photosynthetic Rate (PN) and Stomatal Conductance (gs)

The PN and gs were meticulously recorded at 0, 7, 14, and 21 DWD using a portable photosynthetic system (LI-6400; LICOR, Lincoln, NE, USA). These measurements were conducted on three leaves per replication between 11:00 AM and 1:00 PM.

### 2.5. Chlorophyll Fluorescence (Fv/Fm) and Chlorophyll Color Index (CCI)

Fv/Fm was assessed by recording the photosystem II maximum quantum efficiency, employing a hand-usable Plant Efficiency Analyzer (Hansatech, Norfolk, UK). For this measurement, 30 min. dark adaptation of leaves was carried out by employing clips on the adaxial surface. Excitation irradiance was maintained at 3000 µmol m^−2^ s^−1^. Higher (*F*_0_) and lower (*F_m_*) fluorescence was recorded to calculate Fv/Fm:

*F_v_*/*F_m_* = *F_m_* − *F*_0_/*F_m_*

For CCI measurements, the top three leaves were selected during daytime hours (10:00–11:00 AM) using a handheld chlorophyll meter CCM-200 (Opti-Sciences, Tyngsboro, MA, USA) at wavelengths of 655/940 nm, which are known to be optimal for chlorophyll content determination.

### 2.6. Relative Water Content (RWC) and Electrolyte Leakage (EL)

RWC and EL were measured following the meticulous protocols of Khare [12]. The fresh weight of the leaf samples was measured (FW), and the samples were then immersed in water up to full turgidity for 6 h, and the turgid weight (TW) was measured. Subsequently, the leaf samples were dried at 80 °C using an oven to measure the dry weight (DW). RWC% was measured using the formula below:

RWC% = (FW − DW/TW − DW) × 100

For EL assessment, leaf discs of 1 cm in diameter were taken and dipped in 20 mL of water at room temperature for four hours to measure initial conductivity (EC1). Further discs were autoclaved for 30 min. at 121 °C to measure final conductivity (EC2). Electrolyte leakage was measured using the following formula:

EL% = (EC1/EC2) × 100

### 2.7. Chlorophyll (Chl) and Carotenoid (Car) Measurement

For Chl and Car estimation, approximately 250 mg of leaf tissue was placed in a mortar and pestle and homogenized using acetone (80%). The supernatant absorbance was measured at wavelengths of 663 nm, 645 nm, and 470 nm for Chl a, Chl b, and Car, respectively, on a UV-vis spectrophotometer (UV-1601; Shimadzu, Japan). The pigment concentrations were calculated according to Lichtenthaler and Buschmann [13]:

Chla = [(12.7 × A663) − (2.69 × A645)]

Chlb = [(22.9 × A645) − (4.68 × A663)]

Car = [(1000 × A470) − (3.27 × Chla + Chlb)/227]

### 2.8. Hydrogen Peroxide (H_2_O_2_), Malondialdehyde (MDA), and Proline Content

The estimation of hydrogen peroxide (H_2_O_2_) was carried out using the methodology of Shah [14]. A total of 200 mg was crushed in phosphate buffer (50 mM; pH 6.5), and centrifugation (7500× *g*) was performed for 10 min. The supernatant was collected, mixed with 0.1% titanium sulfate (1 mL), and centrifuged at 7000× *g* for 15 min., and the absorbance was measured at 410 nm utilizing a UV-Vis spectrophotometer (UV-1601; Shimadzu, Japan). Lipid peroxidation in terms of MDA was measured by following Heath and Packer’s protocol [15]. A total of 300 mg of leaf tissues were ground in 3 mL of trichloroacetic acid (TCA) solution (0.1% *w/v*). The mixture was centrifuged (10,000× *g*) for 20 min., and 2 mL of supernatant was mixed with 0.5% (*w/v*) thiobarbituric acid (TBA), followed by incubation for 30 min. Immediately, the reaction was cooled using ice, and centrifugation was performed at 10,000× *g* for 10 min. Absorbance was measured at 532 and 600 nm using a UV-Vis spectrophotometer (UV-1601; Shimadzu, Japan). The proline concentration in the leaf samples was estimated as per Bates’ protocol [16]. A total of 500 mg of fresh leaf tissues was crushed in sulfosalicylic acid, and centrifugation was performed at 10,000× *g* for 10 min. Next, 2 mL of supernatant, acetic acid, and acid ninhydrin were mixed and incubated for 1 h at 100 °C. Furthermore, proline was extracted by adding toluene (4 mL). A UV-Vis spectrophotometer (UV-1601; Shimadzu, Japan) was used to measure the absorbance at 520 nm.

### 2.9. Antioxidant Enzyme Activity Determination

The activity of catalase (EC 1.11.1.6) was determined using the protocol from [17], with minor modifications. Leaf tissues (200 mg) were crushed in Tris NaOH buffer (pH 8.0; 50 mM) with EDTA (0.5 mM), Triton X-100 (0.5% (*v*/*v*)), and PVP (2%) and centrifuged (22,000× *g*) for 15 min., and the supernatant was collected for use as a crude enzyme. The assay mixture comprised 1 mL of KH_2_PO_4_ buffer (pH 7.0; 100 mM), 0.4 mL of H_2_O_2_ (200 mM), and 0.1 mL of the enzyme extract, and the H_2_O_2_ degradation was recorded at 240 nm. To determine SOD (EC 1.15.1.1) activity, we used the detailed methodology from [14]. Leaf tissues (200 mg) were ground in KH_2_PO_4_ buffer (100 mM; pH 7.5), centrifugation was performed for 10 min. (22,000× *g*) at 4 °C, and the supernatant was collected. An assay mixture was prepared consisting of NaHCO_3_ buffer (50 mM; pH 9.8) with EDTA (0.1 mM), epinephrine (0.6 mM), and supernatant in a 3 mL volume reaction.

Absorbance was measured at 470 nm. The activity of ascorbate peroxidase (EC 1.11.1.11) was analyzed as per Nakano and Asada’s [18] methodology. A total of 200 mg of fresh leaf tissues were crushed in KH_2_PO_4_ buffer (50 mM; pH 7.8) with ascorbic acid (1.0 mM), EDTA (1.0 mM), phenylmethylsulfonyl fluoride (1.0 mM), and 1% PVP. An assay was mixed with a volume of3 mL, containing KH_2_PO_4_ buffer (50 Mm; pH 7.0), 0.2 mM EDTA (0.2 mM), ascorbic acid (0.5 mM), H_2_O_2_ (0.2 mM), and the crude enzyme, and the measurement of absorbance was monitored at 290 nm. The activity of glutathione reductase (EC 1.6.4.2) was determined according to the methodology stated in [19]. A total of 200 mg of the sample of fresh leaves was crushed in Tris-HCl buffer (0.1 M; pH 7.8). The2 mL final volume comprised Tris-HCl buffer (100 mM; pH 7.8), NADPH (0.2 mM), oxidized glutathione (0.5 mM), MgCl_2_ (3 mM), and 0.2 mL of the crude enzyme, and the absorbance was noted at 340 nm.

The activity of guaiacol peroxidase (EC 1.11.1.7) was determined by following the Shah methodology [14]. A total of 200 mg of fresh leaf samples were homogenized in 5 mL of NaH_2_PO_4_ (60 mM; pH 7.0), centrifugation was performed at 22,000× *g* for 15 min, and the collected supernatant was used as the crude enzyme in the assay. A reaction mixture was prepared by adding KH_2_PO_4_ buffer (100 mM; pH 6.5), guaiacol (15 mM), H_2_O_2_ (0.05% *v*/*v*/), and 60 µL of the crude enzyme. Guaiacol oxidation was analyzed by an absorbance increment at 470 nm. The absorbance for all the enzymes was recorded with the help of a spectrophotometer (UV-vis 1601 Shimadzu, Japan).

### 2.10. Physiochemical and Nutritional Properties of Soil

The soil’s various physiochemical parameters were measured with the help of well-established methods. Soil pH was measured by a pH meter according to Yan et al. [20], and soil electrical conductivity was determined following Jackson’s methodology, using a conductivity meter as per Jackson et al. [21]. The soil organic carbon (OC) was measured by the K_2_Cr_2_O_7_ oxidation methodology, as described by Salam et al. [22]. Furthermore, the N, P, and K available in the soil were measured by following the protocols of Subbiah and Asija [23], Olsen et al. [24], and Kirkbright and Aargent [25], respectively.

### 2.11. Elemental Analysis

The Na, K, Ca, and Mg concentrations in the root and shoot tissues were estimated with the help of a 7700 × ICP-MS system (Agilent Technologies, Santa Clara, CA, USA). The samples were oven-dried and powdered using a pestle and mortar. The samples were digested, and the Na, K, Ca, and Mg concentrations of the root and shoot tissues were estimated with the help of a 7700 × ICP-MS system (Agilent Technologies, Santa Clara, CA, USA). The samples were oven-dried and powdered using a pestle and mortar. The samples were digested with HNO_3_ (SupraPur™, Merck, Kenilworth, NJ, USA) in a microwave. Subsequently, analysis was performed using methodology of Bhati et al. [26]. Data analysis was performed with the help of Syngistix (version 4.0) for ICP-MS software (PerkinElmer, Waltham, MA, USA). The responses of the signal were normalized to the rhodium (Rh) internal standard, dilution factors, and sample weights [26].

### 2.12. Selection of Genes and Their Expression Analysis

A total of 10 drought-responsive genes were selected for the expression study (Supplementary Table S1). The real-time PCR primers were designed for *APX, CAT, DREB2C, DREB2D, DREB3, dehydrin, GR, POD, RD22*, and *SOD* using Primer3 (v. 0.9) software [27]. RNA extraction from the AHK-200 leaves was carried out using RNeasyPlantMiniKit (Qiagen), as per the manufacturer’s instructions. First-strand cDNA synthesis was conducted using total RNA (1 µg) in a 20 µL volume reaction with the help of a Bio-Rad first-strand cDNA synthesis kit (Bio-Rad, Hercules, CA, USA), as per the manufacturer’s protocol. The analysis of expression was performed with the help of Bio-Rad SsoFastEvaGreenSupermix (Bio-Rad, Hercules, CA, USA) according to the manufacturer’s protocol in an iQ5 thermal cycler (BioRad) with iQ5 software. The qPCR was conducted on a 96-well optical plate. Every reaction consisted of 5 µL of cDNA, 1 µL of the gene specific primer, and 10 µL of SsoFast Eva Green Supermix (Bio-Rad, Hercules, CA, USA). The iQ5 thermal cycler program was set as denaturation at 95 °C for 1 min., followed by 35 cycles at 95 °C for 30 s, at 55–60 °C (varied as per the primer melting temperature) for 30 s, and at 72 °C for 40 s and, finally, one cycle at 72 °C for 5 min. for real-time quantitative PCR. The relative expression of the gene was calculated using the 2^−∆∆CT^ method [28]. ∆∆CT values indicate the relative fold change expression (induction ratio) of the target gene transcription upon water deficit exposure.

### 2.13. Statistical Analysis

Statistical analyses were conducted using SPSS software (Version 16.0, SPSS Inc., Chicago, IL, USA). For each replication, three samples were collected, and the mean value of each replication was used in the statistical analysis. Significance was assessed using a one-way analysis of variance (ANOVA), followed by Duncan’s multiple range test for post hoc analysis. A significance level of 5% (*p* < 0.05) was considered statistically significant.

## 3. Results

The analysis of morphological, physiological, biochemical, and gene expression changes revealed significant alterations across all parameters in response to the varying durations of the water deficit (WD) treatments. The pots’ soil water content (SWC) decreased progressively, measuring 46.1%, 26.8%, 12.5%, and 8.7% at 0, 7, 14, and 21 DWD, respectively.

### 3.1. Soil Parameters

Electrical conductivity (EC) measurements showed no significant changes up to 14 DWD compared to the controls; however, a significant reduction of 13.1% was recorded at 21 DWD relative to the controls. Soil pH remained stable across the treatments, with values around 7.1 for the control conditions and the7 DWD and 21 DWD treatments, while it rose slightly to 7.2 at 14 DWD, indicating non-significant differences among the treatment groups. The organic carbon content in the soil samples showed a slight decrease over time: values were 0.92% in the controls, dropping to 0.91% at 7 DWD, 0.88% at 14 DWD, and 0.87% at 21 DWD, representing decreases of 1.09%, 4.35%, and 5.43% (Table 1).

### 3.2. Morphological Parameters

**Figure 1:**
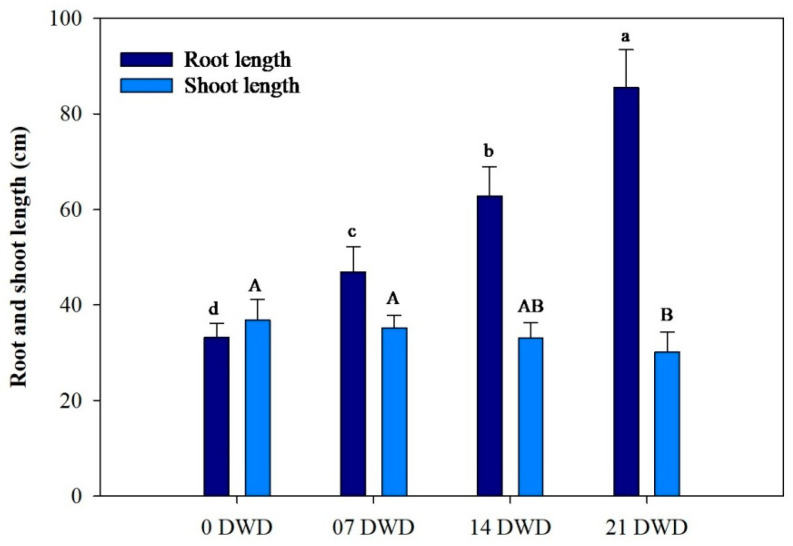
Root and shoot length of AHK-200 plants under 0 (well-watered), 7, 14, and 21 DWD. Means of three replicates ± SEs; values followed by the same letter are not significantly different (*p* ≤ 0.05) according to Duncan’s multiple range test. The small and capital letters correspond to the letters of significance in the corresponding bars.

**Figure 2:**
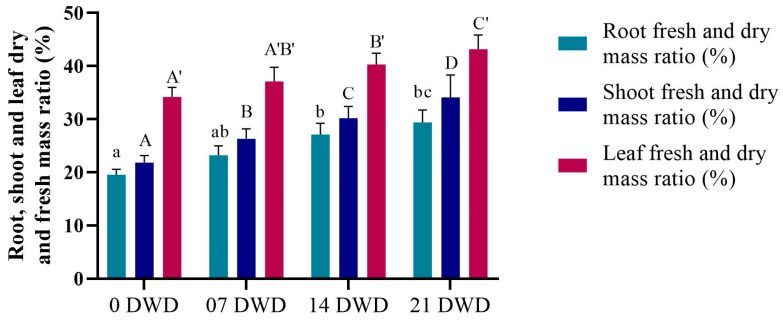
Root, shoot, and leaf dry and fresh mass ratios of AHK-200 plants under 0 (well-watered), 7, 14, and 21 DWD. Means of three replicates ± SEs; values followed by the same letter are not significantly different (*p* ≤ 0.05) according to Duncan’s multiple range test. The small and capital letters with (′) correspond to the letter of significance in the corresponding bars.

As illustrated in Figure 1, root length increased with the increase in the duration of DWD, reaching a maximum of 85.5 cm after 21 DWD, representing increases of 41.26%, 89.15%, and 157.53% compared to the control for the 7, 14, and 21 DWD treatments, respectively, indicating significant growth enhancement under drought conditions. Conversely, shoot length exhibited a slight reduction, decreasing by 4.45%, 10%, and 18% after 7, 14, and 21 DWD compared to the control, while reductions were not statistically significant until after 21 DWD (Figure 1). The ratios of fresh and dry mass for the roots, shoots, and leaves were calculated and showed a significant increase with prolonged DWD exposure. Specifically, the increases in root dry mass (RDM) compared to the control were recorded as 19%, 39%, and 50.8% for the respective DWD periods; shoot dry mass (SDM) increased by 20.64%, 38.53%, and 56.42%, and leaf dry mass (LDM) rose by 8.48%, 17.84%, and 26.32% (Figure 2).

### 3.3. Physiological Parameters

**Figure 3:**
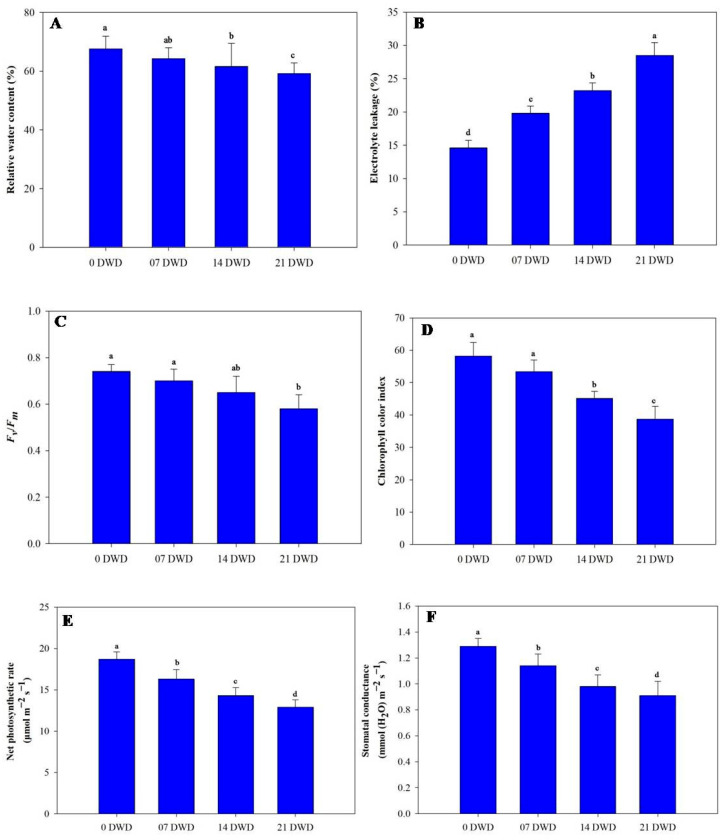
Relative water content (**A**), electrolyte leakage (**B**), *F_v_*/*F_m_* (**C**), chlorophyll color index (**D**), net photosynthetic rate (**E**), and stomatal conductance (**F**) in AHK-200 leaves under 0 (well-watered), 7, 14, and 21 DWD. Means of three replicates ± SEs; values followed by the same letter are not significantly different (*p* ≤ 0.05) according to Duncan’s multiple range test.

With increasing DWD, the relative water content (RWC) exhibited a downward trend; however, reductions were only significant after 21 DWD, where RWC decreased by 4.88%, 8.87%, and 12.42% across the three treatment durations compared to the control plants (Figure 3A). Electrolyte leakage (EL) significantly increased with DWD duration, rising by 35.61%, 58.90%, and 95.20% after the respective treatments (Figure 3B). Photosynthetic efficiency measured as Fv/Fm similarly diminished; while non-significant at a reduction of only 5.45% after 7 DWD, it significantly reduced by 12.2% and 21.6% after the subsequent treatment periods (Figure 3C). The chlorophyll color index (CCI) values were recorded as follows: the control plants had a CCI of 58.2, which dropped to values of 53.4, 45.1, and, finally,38.7 after each respective DWD treatment, indicating reductions of approximately 8.24%, 22.50%, and 33.50% (Figure 3D). The net photosynthetic rate (PN) and stomatal conductance (gs) also declined with increasing DWD; PN decreased by 12.83%, 23.53%, and 31.0% after the three treatment durations (Figure 3E), and gs decreased by corresponding values of 11.63%, 24.0%, and 29.46% (Figure 3F).

### 3.4. Biochemical Parameters

**Figure 4:**
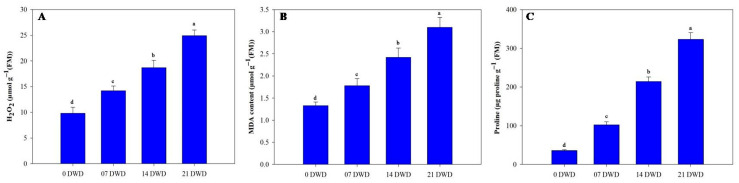
Hydrogen peroxide (H_2_O_2_) (**A**), MDA content (**B**), and proline content (**C**) in AHK-200 leaves under 0 (well-watered), 7, 14, and 21 DWD. Means of three replicates ± SEs; values followed by the same letter are not significantly different (*p* ≤ 0.05) according to Duncan’s multiple range test.

Hydrogen peroxide (H_2_O_2_) concentrations increased under drought stress conditions, rising by approximately 44.90%, 90.8%, and, ultimately, 154% following the respective treatments compared to the controls (Figure 4A). Malondialdehyde (MDA) concentrations varied from a low of 1.33 in the control plants to a peak of 3.1 in plants subjected to prolonged drought stress (Figure 4B). A significant increase in proline concentration was observed under drought conditions; levels rose 2.86-fold after 7 DWD, 6-fold after 14 DWD, and 9-fold following 21 DWD compared to the controls (Figure 4C). Total chlorophyll concentrations showed a non-significant reduction of approximately 6.76% after the first week of drought stress but were significantly reduced by 20.3% and 30.40% after 14 and 21 DWD treatments compared to the controls (Table 2). Carotenoid content also diminished with increasing drought severity; the maximum carotenoid levels were observed in the control plants (1.92 mg g^−1^), while levels dropped to1.48 mg g^−1^ in plants subjected to 21 DWD, indicating reductions of9.90%,17.70%, and 22.90% across the treatment durations (Table 2).

### 3.5. Antioxidant Enzyme Activity

**Figure 5:**
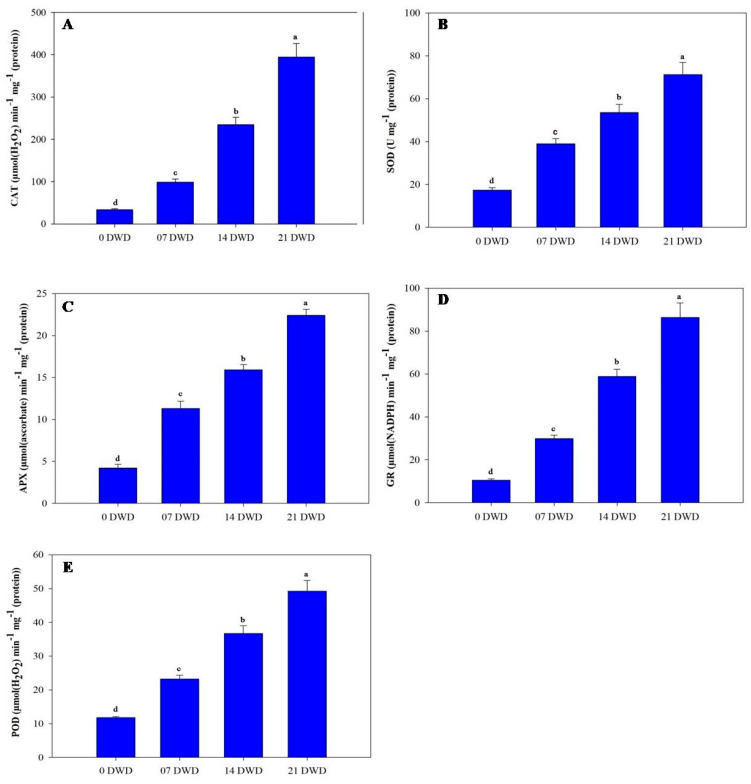
Activities of key antioxidant enzymes in AHK-200 leaves under 0 (well-watered), 7, 14, and 21 DWD. Catalase (CAT) (**A**), superoxide dismutase (SOD) (**B**), ascorbate peroxidase (APX) (**C**), glutathione reductase (GR) (**D**), and guaiacol peroxidase (POD) (**E**). Means of three replicates ± SEs; values followed by the same letter are not significantly different (*p* ≤ 0.05) according to Duncan’s multiple range test.

The activities of antioxidant enzymes such as superoxide dismutase (SOD), catalase (CAT), ascorbate peroxidase (APX), glutathione reductase (GR), and peroxidase (POD) increased significantly with extended drought exposure, peaking at 21 DWD. After 7 DWD, enzyme concentrations rose by 123.56% for SOD, 196.7% for CAT, 169% for APX, 183.8% for GR, and 96.6% for POD; these increases became even more pronounced at 14 DWD, with respective increases of 207.5%, 604.2%, 278.6%, 460%, and 211%. By 21 DWD, further increases were noted of309.2%, 1085%, 433.3%, 721.90%, and 316.95% (Figure 5A–E).

### 3.6. Electrolyte Concentration

Drought stress significantly increased sodium concentrations in leaf tissues, rising by 23.74%, 106.9%, and 213% after 7, 14, and 21 DWD, respectively, compared to the controls. Potassium levels rose significantly during drought exposure; increases were 26.27%, 38.23%, and 52.94% after 7, 14, and 21 DWD, respectively. Calcium concentrations similarly increased significantly, rising by 38.53%, 63%, and 73.25% over the same periods compared to the controls. In contrast, magnesium and iron concentrations decreased significantly under drought stress conditions; magnesium levels dropped by 13.88%, 33.55%, and 50.20% after 7, 14, and 21 DWD, respectively. Iron content exhibited variable responses, increasing by 17.54%, 61.7%, and 48.83% after 7, 14, and 21 DWD, respectively, compared to the controls (Table 3).

### 3.7. Gene Expression Changes

**Figure 6:**
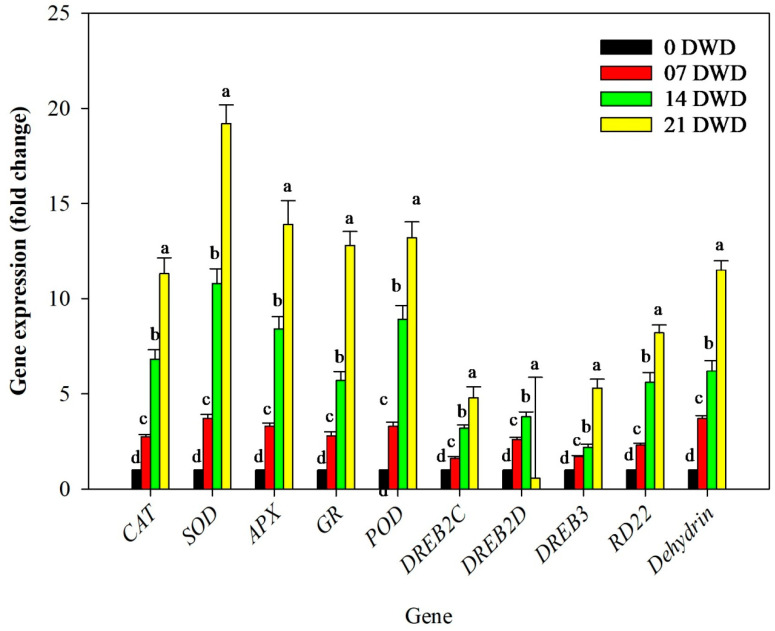
Relative expressions of antioxidative enzyme genes in the leaves of AHK-200 under 7, 14 and 21 DWD: *CAT* (MELO3C017024), *SOD* (MELO3C014007), *APX* (MELO3C013363), *GR* (MELO3C006322), *POD* (MELO3C014652), *DREB2C* (MELO3C008318), *DREB2D* (MELO3C003785), *DREB3* (MELO3C003463), *RD22* (MELO3C003592), and *Dehydrin* (MELO3C016402). Means of three replicates ± SEs; values followed by the same letter are not significantly different (*p* ≤ 0.05).

Drought stress significantly influenced the expression levels of antioxidant enzyme-related genes, including *CAT*, *SOD*, *APX*, *GR*, and *POD*, across all treatment durations. *CAT* expression increased substantially by 2.75-fold after 7 DWD, 6.82-fold after 14 DWD, and 11.32-fold following 21 DWD compared to the controls. *SOD* expression also rose significantly by 3.7-fold after 7 DWD, 10.8-fold after 14 DWD, and 19.2-fold following 21 DWD relative to the control conditions. Similarly, *APX* expression levels increased 3.3-fold after 7 DWD, with even greater increases observed at 14 DWD (8.4-fold) and 21 DWD (13.9-fold). In addition to this, *GR* and *POD* expression rose by 12.8- and 13.2-fold after 21 DWD (Figure 6).

Additionally, *DREB* gene expression levels rose significantly with increasing drought stress; notably, *DREB2C* expression was enhanced by 1.6-fold after 7 DWD, 3.2-fold following 14 DWD, and 4.8-fold after 21 DWD relative to the controls. Similarly, *DREB2D* expression rose significantly in leaf tissues by 2.6-fold, 3.8-fold, and 5.3-fold after 7, 14, and 21 DWD compared to the expression level under control conditions. However, the expression pattern of *DREB3* was random, as it rose by 1.7-fold and 2.2-fold after 7 and 14 DWD compared to the control plants, and it was reduced to 1.9-fold compared to the control expression after 21 DWD (Figure 6)

In addition to this, dehydration-responsible gene *RD22* and *dehydrin* gene expression rose with a rise in the DWD compared to the control plants. The fold increase in gene expression was 2.3, 5.6, and 8.2, respectively, after 7, 14, and 21 DWD in the case of the *RD22* gene and 3.7, 6.2, and 11.5 for the *dehydrin* gene under similar conditions, and the rise was significant (Figure 6). Overall, the findings indicate that prolonged water deficit leads to substantial physiological adaptations in AHK-200 through morphological changes, enhanced antioxidant activity, alterations in biochemical parameters, shifts in soil properties, electrolyte balance adjustments, and significant gene expression modifications related to stress tolerance mechanisms.

## 4. Discussion

This is the first time we have carried out an elaborate study to expand our knowledge regarding the mechanism behind the higher drought tolerance potential of the AHK-200 genotype. Breeders are being forced to implement this genotype to develop drought-tolerant genotypes belonging to cucurbits and other related crops. In addition, this gene expression study will highlight the role of these genes in AHK-200 drought tolerance, which can further assist in gene manipulation, gene utilization, transgenic development, and designing CRISPR-based experiments to develop drought-tolerant genotypes in cucurbits and other related crops with better yield potential.

In general, drought stress resulted in increased root length. Plant roots are the key organ for water and nutrient absorption, which is necessary for the productivity of crops, particularly under drought stress [29]. The root length is a morphological feature that better explains the root’s capability to explore nutrient and moisture absorption from the soil’s deeper layer [30]. An increment in the length of the root was noted under progressive WD stages in the present study, with maximum rises after 21 DWD; however, the increase in root length of the AHK-200 genotype compared to the control was much higher than in our earlier reports on muskmelon [11] and snapmelon [31]. This suggests that the higher tolerance to drought stress by AHK-200 might be attributed to a better-penetrating root length, which assists in the absorption of water from the soil’s deeper layer.

Factually, a deeper and more vigorous root was reported to be linked with improved drought tolerance in plants [11]. Better water-holding capacity, which is described in terms of the fresh-to-dry mass ratio, is one of the important factors responsible for the better tolerance of crops to WD, as is evident from our earlier reports on muskmelon and snapmelon [11,31], also reported by another group [32] and observed in the present study in the case of AHK-200. Plants displaying higher WD tolerance displayed higher water-withholding potential, which is evident from the enhanced dry-to-fresh biomass ratio of tissues like leaves, shoots, and roots under WD conditions. According to Patanè et al. [33], a relative increment in the fresh-to-dry mass ratio in organs such as leaves, roots, and shoots could act as an osmotic stress balance indicator under WD conditions. The RWC is the measurement of tissues’ maximum water-holding capacity and displays the most suitable water status measurement in plants in the form of physiological changes at the cellular level under drought stress [34].

The RWC in AHK-200 lowered as DWD was enhanced; although the RWC reduction was non-significant up to 14 DWD, a slight and significant reduction was observed after 21 DWD. The better water-retaining capacity of AHK-200, even under 21 DWD, might be a noticeable physiological adaptation that makes this genotype more tolerant to drought stress. However, in the case of muskmelon and snap melon, a significant and higher reduction in RWC was earlier reported by our groups [11,31,32], which also reported an RWC decrease under deficit irrigation in *Cucumis melo*, similar to our findings. Cell membrane stability is assessed by EL measurements; low EL level is an indicator of high stability in the membrane and higher tolerance to water stress. The plants exposed to WD for a long duration showed harsh damage to the membrane, resulting in increased EL and ion loss; EL and ions play a pivotal role in the functioning of cells. AHK-200 showed much lower EL levels, even after 21 DWD, indicating AHK-200 has higher membrane stability under WD stress; hence, it was identified as a drought-tolerant genotype.

Drought stress causes a significant reduction in *PN* [35]. This could be because of the abscisic acid-induced closure of the stomata and low CO_2_ availability, leading to reduced activity of the photosynthetic apparatus [36]. The reduction in PN was linked with photosynthetic machinery damage and a reduction in gs; however, photosynthetic machinery damage caused by WD was minimal in AHK-200 in comparison to our earlier reports [11,31]. Together with PN, the higher gs in AHK-200 suggests it has better WD tolerance ability since it is well established that enhanced gs helps CO_2_ influx for photosynthesis in leaves. Chl fluorescence in terms of *F_v_*/*F_m_* gives insights into the degree of injury under stress to the photosynthetic components. WD causes a negative alteration in the PSII reaction center [37]; in response to WD in AHK-200, photoinhibition was not very pronounced, which showed it has reduced photoinhibition with increasing WD. PSII efficiency reduction in watermelon was also measured when plants were exposed to different degrees of water stress [38]. In our earlier reports on muskmelon and snap melon, we found a higher reduction in photosynthetic efficiency than in AHK-200, which suggests AHK-200’s better tolerance potential contributed to better photosynthetic efficiency, even under 21 DWD [11,31].

WD leads to damage to the structure of chloroplast and lowers the biosynthesis of Chl [11], and the total Chl content of AHK-200 decreases as WD progresses. Although the reduction was lower than that in our earlier reports on muskmelon and snap melon [11,31], the relatively high Chl content in AHK-200 displayed a higher capability of AHK-200 to tolerate WD than muskmelon and snap melon crops. In the same way, a decrease was reported in the content of Car with an increase in DWD. Such a decrease might be because of the degradation of Car or a lower biosynthesis of Car. However, the Car concentration was high in AHK-200 compared to the muskmelon and snap melon crops in our earlier reports [11,31], which suggests a better tolerance potential in this genotype. Soil factors like EC, pH, and OC did not change much with the rise in DWD, and most of the parameters did not change significantly up to 21 DWD, except EC after 21 DWD. Non-significant changes in soil parameters might have contributed toward the better adaptive potential of AHK-200 to resist better, even under 21 DWD, and suggest that these factors are involved in the higher tolerance potential of AHK-200 to drought stress.

The presence of H_2_O_2_ is considered indicative of ROS. Furthermore, excess H_2_O_2_ accumulation causes oxidative stress in plant cells at higher concentrations [39]. In this experiment, we found enhanced accumulation of H_2_O_2_ with a rise in DWD in AHK-200, although the rise was lower than that in our earlier report on muskmelon and snap melon [11,31]. The balanced ROS production in AHK-200 might contribute toward better tolerance in AHK-200 [38,40]. MDA is produced by lipid peroxidation and is considered an indicator of oxidative damage to the membrane caused by stress. Previous reports pointed out that MDA decreased upregulation is linked to drought stress tolerance in muskmelon [41]. Although the rise in the level of MDA recorded in the current study was at its maximum after 21 DWD, it was less concerning than in earlier reports [11,31]. In the same way, the increment in MDA content under drought stress was reported to be lower in wild watermelon plants (*Citrullus lanatus* var. *Citrode*), which are more drought-tolerant than cultivated sensitive varieties (*Citrullus lanatus* var. *lanatus*) [38]. Increased proline concentration with the increase in DWD is important because of its action as a compatible solute and protector of protein and membrane integrity and as a ROS scavenger, maintaining the cellular redox status [42], and it acts as an Osmo protectant. In the case of AHK-200, the rise in proline levels was much higher with the rise in DWD, and this is higher than in earlier reports on muskmelon and snap melon [11,31]. This might be a factor in the greater drought adaptability of AHK-200.

SOD is one of the ubiquitous enzymes and plays a significant function in ROS detoxification under oxidative stress. Elevated SOD activity in muskmelon [11] and snap melon [31] is found under drought stress. A higher SOD activity in AHK-200 also corresponds with its better tolerance to oxidative damage under drought stress. CAT also displayed increased activity in AHK-200, but it was comparatively higher than in muskmelon and snap melon [11,31], suggesting more effective H_2_O_2_ scavenging in AHK-200. Enhanced CAT activities under drought stress were reported in cucumber and muskmelon by Qian et al. [43] and Kusvuran [41], respectively. In this study, elevated APX and GR activities in AHK-200 corroborate our earlier reports on muskmelon and snap melon, as a more tolerant genotype showed a higher level of APX and GR, although AHK-200 showed a higher increase in the level of these enzymes, making it a more adaptive genotype to drought stress. The increased activity of POD has also been found in different crops, such as common bean, sunflower, and sorghum [44], muskmelon and snap melon [11,31], and tomato [45], under drought stress. Similarly, enhanced POD activity with the rise in DWD in AHK-200 suggests the contribution of this enzyme to the better tolerance of AHK-200 to drought stress.

The expression of *APX, Cu/Zn-SOD*, and *Mn-SOD* transgenes improves drought tolerance in plants [46]. Our study resulted in similar results; *APX, CAT*, and *SOD* expressions were elevated with increasing DWD, and upregulation was higher after 21 DWD, proving the significance of these genes in the drought tolerance of AHK-200. In our earlier reports, we found higher expression of these genes, including the *GR* gene, in the SC-15 water-deficit-tolerant genotype, which increased with the rise in DWD [42]. It is well established that oxidative damage induced by osmotic stresses can be minimized by antioxidant enzyme activation and osmolyte biosynthesis for ROS conversion into less toxic products. This is also evident in the current study with the rise in DWD enzyme activity, which also appeared in the expression pattern of the respective genes. Similar to POD activity, in the case of AHK-200, the expression of *POD* was upregulated with the rise in DWD, and it was at its highest after 21 DWD. Higher expression of the *POD* gene was also observed in the muskmelon [42] and snap melon [31], and the enhanced expression of this gene in AHK-200 could be the reason for its higher tolerance to drought stress. In this study we noted a continuous increment in the expression of the *DREB2C* gene even after 21 DWD, which is contrary to our earlier reports in the case of muskmelon, where the expression of this gene was reduced after 14 DWD [42]. However, in our study, enhanced expression of *DREB2C* suggests the role of this gene in better drought tolerance potential to drought stress. The expression of *DREB2D* was significantly higher until 21 DWD, which is a little contrary to our earlier reports on muskmelon, in which enhanced expression was noted in the drought-tolerant genotype after 7 DWD, with a reduction after 14 DWD and an increase again in expression after 21 DWD, although in the susceptible genotype of muskmelon EC-564755, the expression increased until 7 DWD and then decreased [42]. The continuously enhanced expression pattern in AHK-200 suggests the involvement of this gene in the better drought stress tolerance of this genotype. *DREB3* expression rose until 14 DWD, and then a lower expression of this gene was reported in AHK-200. This is contrary to our earlier reports, in which we found a downregulation in the expression of this gene in the drought-tolerant genotype SC-15 [42]. However, it was earlier reported by Hackenberg et al. [47] that the overexpression of *DREB3* improved drought tolerance in transgenic barley. The binding of *AtMYC2* and *AtMYB2* with the *cis*-acting element of the *rd22* promoter activates *RD22*, as reported by Shinozaki and Yamaguchi-Shinozaki [48]. After 7, 14, and 21 DWD, *RD22* exhibited sustained overexpression in AHK-200. Earlier, it was reported that *RD22* expression was induced in transgenic cotton against drought stress [49]. *Dehydrins* are predominantly upregulated in response to abiotic stresses, like cold, drought, and salinity [42]. As DWD increased, *dehydrin* expression also increased in the AHK-200 genotype. The *dehydrin* elevated expression against drought stress suggests that it plays a functional role in AHK-200-genotype drought tolerance.

Na, K, and Ca concentrations rose, with a maximum increase after 21 DWD in the present study. Soybean genotypes exhibiting a delay-wilting feature against drought stress had a higher accumulation of Ca, Na, and K. This could be linked to the uptake of Na, K, and Ca, which play an important function in cellular homeostasis [50]. Potassium has a positive correlation with plants’ physiological properties, like the efficiency of water utilization, stomatal movement control, and underground and aerial biomass, and it also has an important function in photosynthesis, as reported by Sardans et al. [51]. In taller plants, K is an essential element, as it works as a regulator of osmotic and turgor pressure. It plays a crucial role in stomatal closing and opening and plant growth attributes. The exogenous application of K in maize improved tolerance against drought by controlling physiological and molecular processes such as osmotic regulation, energy, pressure balance, and the synthesis of proteins [52]. Against drought stress, the efficiency of water use was altered in AHK-200 leaves; higher K and Na concentrations enhanced starch and influenced total soluble sugar parallelly in the leaf [53]. When the K level was inadequate, Na positively regulated the biochemical and physiological responses. Ca ions work as a secondary messenger, activating basic physiological phenomena in cells against drought stress, and are essential for almost every stage of plant life. Ca also plays an important role in the growth and regulation of polar cells and tissues as well as adaptation to stress. Ca also regulates stomatal closing and plant adaptation to drought stress mediated by abscisic acid (ABA)-induced effects [54]. Higher concentrations with an increase in DWD suggest the better adaptive potential of AHK-200 to drought stress. The concentration of Mg was significantly affected in AHK-200 under drought stress. As DWD increased, the Mg concentration decreased in AHK-200 leaves. Previously, under drought stress, a decrease in Mg concentrations in tomatoes was reported by Nahar and Gretzmacher [55]. In safflower (*Carthamus tinctorius*), K enhanced the leaf’s relative moisture content; however, Mg decreased it [56]. Mg application has a potential impact on chlorophyll in leaves and a negative impact on the Ca content of safflower [56]. Urbina et al. [57] reported an increment in the concentration of Fe against drought, which confirms the findings of this study. Fe is a mandatory component in chlorophyll, enzyme systems, photosynthesis, and plant respiration [58]. An increase in Fe content in AHK-200 leaves was recorded, and it was enhanced with DWD increment, with the highest value after 21 DWD.

## 5. Conclusions

This study highlights the exceptional adaptability of the AHK-200 genotype, which demonstrates superior morphological, physiological, biochemical, and molecular traits that contribute to its resilience under water deficit conditions. The findings indicate that AHK-200 exhibits enhanced root development, improved water retention capacity, and greater membrane stability than other related melon species, such as muskmelon and snap melon. Moreover, the research underscores the importance of specific biochemical factors and gene expression patterns in mediating drought tolerance. The upregulation of antioxidant enzymes and Osmo protectants, alongside a robust response in genes associated with stress tolerance, positions AHK-200 as a highly promising candidate for breeding programs to develop drought-resistant cultivars. The ability of AHK-200 to maintain photosynthetic efficiency and chlorophyll content under drought stress further emphasizes its potential for cultivation in arid regions, inspiring future research and breeding efforts. In conclusion, the comprehensive analysis of vegetable melon demonstrates that leveraging genetic diversity and understanding the underlying mechanisms of drought tolerance can significantly enhance crop resilience in challenging environments. This study paves the way for future breeding efforts, offering the hopeful prospect of integrating these traits into new varieties to improve yield stability and food security in hot, arid climates. The potential impact of this research on sustainable agriculture is promising, setting a foundation for future research to optimize germplasm utilization in drought-prone areas.

## Figures and Tables

**Table 1:** Effect of increasing days of water deficit (DWD) on electrical conductivity (EC), pH, and organic carbon content in AHK-200.

Treatments	EC (Mosiemens/cm)	pH	Organic Carbon (%)
0 DWD	198 ± 7.38 ^a^	7.1 ± 0.28 ^a^	0.92 ± 0.11 ^a^
07 DWD	192 ± 11.25 ^a^	7.1 ± 0.49 ^a^	0.91 ± 0.08 ^a^
14 DWD	185 ± 6.32 ^ab^	7.2 ± 0.52 ^a^	0.88 ± 0.05 ^a^
21 DWD	172 ± 10.44 ^c^	7.1 ± 0.22 ^a^	0.87 ± 0.09 ^a^

Note: Data are means ± SE of three replications. Means followed by the same letter within a column are not significantly different (*p* > 0.05) according to Duncan’s multiple range tests. DWD represents days of water deficit treatment for the respective day.

**Table 2:** Effect of increasing water deficit on total chlorophyll and carotenoid content in AHK-200.

Treatments	Total Chlorophyll (mg g^−1^ (DM))	Carotenoid (mg g^−1^ (DM))
0 DWD	59.2 ± 7.38 ^a^	1.92 ± 0.09 ^a^
07 DWD	55.1 ± 11.25 ^a^	1.73 ± 0.11 ^b^
14 DWD	47.4 ± 6.32 ^b^	1.58 ± 0.08 ^c^
21 DWD	41.0 ± 10.44 ^c^	1.48 ± 0.07 ^d^

Note: Data are means ± SE of three replications. Means followed by the same letter within a column are not significantly different (*p* > 0.05) according to Duncan’s multiple range tests. DWD represents days of water deficit treatment for the respective day.

**Table 3:** Effect of increasing water deficit on Na, K, Mg, Fe, and Ca concentrations in AHK-200.

Treatments	Na (µg/g)	K (µg/g)	Mg (µg/g)	Fe (µg/g)	Ca (µg/g)
0 DWD	1108 ± 63.2 ^cd^	34 ± 2.43 ^c^	2024 ± 117.2 ^a^	342.2 ± 12.1 ^d^	314.4 ± 18.2 ^d^
07 DWD	1371 ± 82.4 ^c^	43 ± 3.22 ^b^	1743 ± 73.2 ^b^	402.1 ± 27.3 ^c^	435.2 ± 23.9 ^c^
14 DWD	2292 ± 143.2 ^b^	47 ± 2.53 ^ab^	1345 ± 88.7 ^c^	553.3 ± 44.2 ^b^	512.3 ± 36.7 ^b^
21 DWD	3473 ± 182.2 ^a^	52 ± 3.32 ^a^	1008 ± 24.1 ^d^	509.7 ± 21.9 ^a^	544.2 ± 17.8 ^a^

Note: Data are means ± SE of three replications. Means followed by the same letter within a column are not significantly different (*p* >0.05) according to Duncan’s multiple range tests. DWD represents days of water deficit treatment for the respective day.

## Data Availability

All the data recorded during the experiments are presented in the manuscript.

## Supplementary Materials

The following supporting information can be downloaded at: https://www.mdpi.com/article/10.3390/metabo15060359/s1, Table S1: DNA sequences of PCR primers used in real-time quantitative PCR.

## References

[1] Ansari W.A., Atri N., Yang L., Singh B., Pandey S. (2020). Genetic diversity in muskmelon based on SSR markers and morphological traits under well-watered and water-deficit condition. Biocatal. Agric. Biotechnol..

[2] Pandey S., Ansari W.A., Jha A., Bhatt K.V., Singh B. (2011). Evaluation of melons and indigenous *Cucumis* spp. genotypes for drought tolerance. II Int. Symp. Underutilized Plant Species Crops Future-Beyond FoodSecur..

[3] Samadia D.K., Haldhar S.M., Ram H., Verma A.K., Gurjar P.S. (2024). Kachri melon (a non-dessert form of *Cucumis melo*) diversity, germplasm utilization and varietal development under hot arid climate: Approaches and realization. J. Agric. Ecol..

[4] Samadia D.K., Haldhar S.M. (2018). Strategies and advancements for improvement in arid vegetables. Indian J. Arid. Hortic..

[5] Samadia D.K., Haldhar S.M. (2020). Mateera, watermelon (*Citrullus lanatus*) germplasm utilization for improving fruit quality and marketable harvest under hot arid climate of India: Approaches and out-put. J. Agric. Ecol..

[6] Samadia D.K. (2007). Studies on genetic variability and scope of improvement in round melon under hot arid conditions. Indian J. Arid. Hortic..

[7] Samadia D.K., Haldhar S.M. (2019). Scope and strategies for genetic improvement in vegetable crop-plants under high temperature and abiotic stressed climate of Rajasthan: A gap analysis. J. Agric. Ecol..

[8] Haldhar S.M., Samadia D.K., Bhargava R., Choudhary B.R., Singh D. (2018). Host plant accessions determine bottom-up effect of snapmelon (*Cucumis melo* var. *momordica*) against melon fly (*Bactroceracucurbitae* (Coquillett)). Breed. Sci..

[9] Samadia D.K., Pareek O.P. Kachari: Arid region cucurbit vegetable for processing industry. Proceedings of the 4th Agricultural Science Congress.

[10] Kausar A., Ashraf M.Y., Ali I., Niaz M., Abbass Q. (2012). Evaluation of sorghum varieties/lines for salt tolerance using physiological indices as screening tool. Pak. J. Bot..

[11] Ansari W.A., Atri N., Singh B., Kumar P., Pandey S. (2018). Morpho-physiological and biochemical responses of muskmelon genotypes to different degree of water deficit. Photosynthetica.

[12] Khare N., Goyary D., Singh N.K., Shah P., Rathore M., Anandhan S., Sharma D., Arif M., Ahmed Z. (2010). Transgenic tomato cv. Pusa Uphar expressing a bacterial mannitol-1-phosphate dehydrogenase gene confers abiotic stress tolerance. Plant Cell Tissue Organ Cult..

[13] Lichtenthaler H.K., Buschmann C. (2001). Chlorophylls and carotenoids: Measurement and characterization by UVVIS spectroscopy. Curr. Protoc. Food Anal. Chem..

[14] Shah K., Kumar R.G., Verma S., Dubey R.S. (2001). Effect of cadmium on lipid peroxidation, superoxide anion generation and activities of antioxidant enzymes in growing rice seedlings. Plant Sci..

[15] Heath R.L., Packer L. (1968). Photoperoxidation in Isolated Chloroplasts. I. Kinetics and Stoichiometry of Fatty Acid Peroxidation. Arch. Biochem. Biophys..

[16] Bates L.S., Waldren R.P.A., Teare I.D. (1973). Rapid determination of free proline for water-stress studies. Plant Soil..

[17] Rai A.C., Singh M., Shah K. (2012). Effect of water withdrawal on formation of free radical, proline accumulation and activities of antioxidant enzymes in ZAT12- transformed transgenic tomato plants. Plant Physiol. Bioch..

[18] Nakano Y., Asada K. (1981). Hydrogen peroxide is scavenged by ascorbate specific peroxides in spinach chloroplast. Plant Cell Physiol..

[19] Sánchez-Rodríguez E., Rubio-Wilhelmi M., Cervilla L.M., Blasco B., Rios J.J., Rosales M.A., Romero L., Ruiz J.M. (2010). Genotypic differences in some physiological parameters symptomatic for oxidative stress under moderate drought in tomato plants. Plant Sci..

[20] Yan F., Schubert S., Mengel K. (1996). Soil pH changes during legume growth and application of plant material. Biol. Fertil. Soils.

[21] Jackson M.L. (1973). Soil Chemical Analysis.

[22] Salam A.K., Desvia Y., Sutanto E., Syam T., Nugroho S.G., Kimura M. (1999). Activities of soil enzymes in different land-use systems in middle terrace areas of Lampung Province, South Sumatra, Indonesia. Soil Sci. Plant Nutri..

[23] Subbiah B.V., Asija G.L. (1956). A Rapid Procedure for the Estimation of Available Nitrogen in Soils. Curr. Sci..

[24] Olsen S.R. (1954). Estimation of Available Phosphorus in Soils by Extraction with Sodium Bicarbonate (No. 939).

[25] Kirkbright G.F., Sargent M. (1974). Atomic Absorption and Fluorescence Spectroscopy.

[26] Bhati K.K., Aggarwal S., Sharma S., Mantri S., Singh S.P., Bhalla S., Kaur J., Tiwari S., Roy J.K., Tuli R. (2014). Differential expression of structural genes for the late phase of phytic acid biosynthesis in developing seeds of wheat (*Triticum aestivum* L.). Plant Sci..

[27] Rozen S., Skaletsky H. (2000). Primer3 on the WWW for general users and for biologist programmers. Methods Mol. Biol..

[28] Livak K.J., Schmittgen T.D. (2001). Analysis of relative gene expression data using real-time quantitative PCR and the 2^−ΔΔ^CT method. Methods.

[29] Vadez V. (2014). Root hydraulics: The forgotten side of roots in drought adaptation. Field Crops Res..

[30] Siddiqui M.N., Léon J., Naz A.A., Ballvora A. (2021). Genetics and genomics of root system variation in adaptation to drought stress in cereal crops. J. Exp. Bot..

[31] Ansari W.A., Krishna R., Yadav P.S., Chaubey T., Behera T.K., Bhat K.V., Pandey S. (2024). Alteration in physio-chemical properties and gene expression pattern of snapmelon (*Cucumis melo* var. *momordica*) genotypes against drought stress. Plant Genet. Res..

[32] Mirabad A.A., Lotfi M., Roozban M.R. (2013). Impact of water-deficit stress on growth, yield and sugar content of cantaloupe (*Cucumis melo* L.). Proc. Int. Conf..

[33] Patanè C., Scordia D., Testa G., Cosentino S.L. (2016). Physiological screening for drought tolerance in Mediterranean long-storage tomato. Plant Sci..

[34] Khoyerdi F.F., Shamshiri M.H., Estaji A. (2016). Changes in some physiological and osmotic parameters of several pistachio genotypes under drought stress. Sci. Hortic..

[35] Gill S.S., Tuteja N. (2010). Reactive oxygen species and antioxidant machinery in abiotic stress tolerance in crop plants. Plant Physiol. Biochem..

[36] Khazaei Z., Esmaielpour B., Estaji A. (2020). Ameliorative effects of ascorbic acid on tolerance to drought stress on pepper (*Capsicum annuum* L.) plants. Physiol. Mol. Biol. Plants.

[37] Fraga H., Moriondo M., Leolini L., Santos J.A. (2020). Mediterranean olive orchards under climate change: A review of future impacts and adaptation strategies. Agronomy.

[38] Kurtar E.S., Seymen M., Yavuz D., Acar B., Metin D., Atakul Z., Kal Ü. (2024). Morphophysiological and biochemical investigation of the potential of citron watermelon (*Citrullus lanatus* var. *citroides*) rootstock under different irrigation regimes. Hortic. Environ. Biotechnol..

[39] Deeba F., Pandey A.K., Ranjan S., Mishra A., Singh R., Sharma Y.K., Shirke P.A., Pandey V. (2012). Physiological and proteomic responses of cotton (*Gossypium herbaceum* L.) to drought stress. Plant Physiol. Biochem..

[40] Koç C., Ulusu F., Ulusu Y. (2024). Physio-biochemical responses of registered bread wheat (*Triticum aestivum* L.) genotypes to drought stress: Variations in antioxidant parameters and photosynthetic pigment amounts. Anatol. J. Bot..

[41] Kusvuran S. (2012). Effects of drought and salt stresses on growth, stomatal conductance, leaf water and osmotic potentials of melon genotypes (*Cucumis melo* L.). Afr. J. Agric. Res..

[42] Ansari W.A., Atri N., Singh B., Pandey S. (2017). Changes in antioxidant enzyme activities and gene expression in two muskmelon genotypes under progressive water stress. Biol. Plantarum.

[43] Qian C.L., Zhao Y.Y., Mi H.B., Chen X.H., Guo L.J., Mao L.C. (2012). Role of antioxidative system during the development and senescence of cucumber fruit. Biol. Plantarum.

[44] Cook R., Lupette J., Benning C. (2021). The role of chloroplast membrane lipid metabolism in plant environmental responses. Cells.

[45] Krishna R., Ansari W.A., Jaiswal D.K., Singh A.K., Prasad R., Verma J.P., Singh M. (2021). Overexpression of *AtDREB1* and *BcZAT12* genes confers drought tolerance by reducing oxidative stress in double transgenic tomato (*Solanum lycopersicum* L.). Plant Cell Rep..

[46] Mishra N., Jiang C., Chen L., Paul A., Chatterjee A., Shen G. (2023). Achieving abiotic stress tolerance in plants through antioxidative defense mechanisms. Front. Plant Sci..

[47] Hackenberg M., Shi B.J., Gustafson P., Langridge P. (2012). A transgenic transcription factor (TaDREB3) in barley affects the expression of microRNAs and other small non-coding RNAs. PLoS ONE.

[48] Shinozaki K., Yamaguchi-Shinozaki K. (2007). Gene networks involved in drought stress response and tolerance. J. Exp. Bot..

[49] Yue Y., Zhang M., Zhang J., Tian X., Duan L., Li Z. (2012). Overexpression of the *AtLOS5* gene increased abscisic acid level and drought tolerance in transgenic cotton. J. Exp. Bot..

[50] Bellaloui N., Turley R.B. (2013). Effects of fuzzless cottonseed phenotype on cottonseed nutrient composition in near isogenic cotton (*Gossypium hirsutum* L.) mutant lines under well-watered and water stress conditions. Front. Plant Sci..

[51] Sardans J., Grau O., Chen H.Y., Janssens I.A., Ciais P., Piao S., Peñuelas J. (2017). Changes in nutrient concentrations of leaves and roots in response to global change factors. Glob. Change Biol..

[52] Nikju M.B., Mobasser H.R., Ganjali H.R. (2015). Influence of variety on biological yield, harvest index, percent of protein in *Zea mays*. Biol. Forum.

[53] Tadayyon A., Nikneshan P., Pessarakli M. (2018). Effects of drought stress on concentration of macro- and micro-nutrients in Castor (*Ricinus communis* L.) plant. J. Plant Nutr..

[54] Liu H., Song S., Zhang H., Li Y., Niu L., Zhang J., Wang W. (2022). Signaling transduction of ABA, ROS, and Ca^2+^ in plant stomatal closure in response to drought. Int. J. Mol. Sci..

[55] Nahar K., Gretzmacher R. (2011). Response of shoot and root development of seven tomato cultivars in hydrophonic system under water stress. Acad. J. Plant Sci..

[56] Vafaie A., Ebadi A., Rastgou B., Moghadam S.H. (2013). The effects of potassium and magnesium on yield and some physiological traits of safflower (*Carthamus tinctorius*). Int. J. Agric. Crop Sci..

[57] Urbina I., Sardans J., Beierkuhnlein C., Jentsch A., Backhaus S., Grant K., Peñuelas J. (2015). Shifts in the elemental composition of plants during a very severe drought. Environ. Exp. Bot..

[58] Afshar R.M., Hadi H., Pirzad A. (2013). Effect of nano-iron on the yield and yield component of cowpea (*Vigna unguiculata*) under end season water deficit. Int. J. Agric..

