# Supplementary material for: Physiological and Molecular Responses of Underutilized Genotype AHK-200 of Vegetable Melon (Cucumis melo var. melo) Against Drought Stress: Gas Exchange, Antioxidant Activity, and Gene Expression
Source: Metabolites. 2025 May 28;15(6):359. doi: 10.3390/metabo15060359 (PMC12195343; doi:10.3390/metabo15060359)
Supplement: Supplementary file 1 [file metabolites-15-00359-s001.zip › metabolites-3608510-supplementary.pdf]

Table S1. DNA sequences of PCR primers used in real-time quantitative PCR.

Unigene ID	Genes	Forward Primer Sequence	Reverse Primer Sequence
MELO3C017024	*CAT*	CTCTGCCTTGACCATTGGAT	AGCATGAACAACACGCTCTG
MELO3C014007	*SOD*	CCATCCAGTTCGTCCAAGAT	GTATCGCCAAGAGCATGGAT
MELO3C013363	*APX*	CTGGTTTTGATGGACCTTGG	ACATATTGGCGGAACTCAGG
MELO3C006322	*GR*	AGAGAAAGCGAAGGGTGACA	GCCAAGGACCTTTTGTGTGT
MELO3C014652	*POD*	CCTCCAAAGAATCCGTCGTA	TTGGCTTTGAGTGCATTGAG
MELO3C008318	*DREB2C*	TACTTGGGGCTGCGTAATTC	TCCTCGATTCTCCTTCTCCA
MELO3C003785	*DREB2D*	ATGAAGCCGCTCTTGCTTAC	ATCGGAGATGAGGACATCGT
MELO3C003463	*DREB3*	CGATAACAACAGCAGCAGCA	TGAGCGAGTTCAGGGAAGTT
MELO3C003592	*RD22*	AAACCGGGCTACAGTCCTTT	GTGGTGGTGGTTGTTGTTTG
MELO3C016402	*Dehydrin*	CGCATTACCAATCTGGAGGA	TTCCAACTGGGTTACCGAAC
AY859055	*Actin*	CCCTGGTATTGCAGACAGGA	ACATCTGCTGGAAGGTGCTT
